# Antioxidant, Anti-Inflammatory, and Antitumoral Effects of Aqueous Ethanolic Extract from* Phoenix dactylifera* L. Parthenocarpic Dates

**DOI:** 10.1155/2018/1542602

**Published:** 2018-08-06

**Authors:** Hanen El Abed, Mouna Chakroun, Zaineb Abdelkafi-Koubaa, Noureddine Drira, Naziha Marrakchi, Hafedh Mejdoub, Bassem Khemakhem

**Affiliations:** ^1^Laboratory of Plant Biotechnology, Sfax Faculty of Sciences, BP 1171, University of Sfax, 3038 Sfax, Tunisia; ^2^Laboratory of Venoms and Therapeutic Biomolecules (R11IPT08), Institut Pasteur of Tunis, 13 Place Pasteur, 1002 Tunis, Tunisia; ^3^University of Tunis el Manar, 1068 Tunis, Tunisia

## Abstract

The aim of this study was to evaluate the antioxidant, the anti-inflammatory, and the antitumoral activities of the aqueous ethanolic extract from* Phoenix dactylifera* L. parthenocarpic dates. The antioxidant activity was carried using DPPH radical scavenging activity. The result showed that parthenocarpic dates had strongly scavenging activity on DPPH reaching 94% with an IC_50_ value of 0.15 ± 0.011 mg/mL (p < 0.05). The anti-inflammatory potential was determined by the inhibitory effect of the aqueous ethanolic extract on phospholipase A_2_ activity as well as on carrageenan-induced paw oedema in mice. The* in vitro* study showed that the extract inhibited the phospholipase A_2_ activity with an IC_50_ value of 130 *μ*g/mL and the* in vivo* study showed a significantly decrease in the paw oedema after 1 h compared to the control group. Finally, the antiproliferative activity of the aqueous ethanolic extract was assessed by MTT test against MCF-7 and MDA-MB-231 cancer cell lines. This extract was effective in inhibiting MDA-MB-231 and MCF-7 cancer cells growth with IC_50_ values of 8 and 18 mg/mL, respectively, after 72 h treatment. These results confirm the ethnopharmacological significance of* Phoenix dactylifera* L. parthenocarpic dates, which could add support for its pharmaceutical use.

## 1. Introduction

Oxidative stress is an important risk factor in the pathogenesis of numerous chronic diseases. Free radicals and other reactive oxygen species can adversely affect various important classes of biological molecules, such as protein, deoxyribonucleic acid (DNA), and lipids causing oxidative deterioration of biomolecules [1]. This damage can lead to various human diseases, especially aging, heart disease, stroke, arteriosclerosis, diabetes, cancer, and inflammation [1].

Inflammation is considered as a primary physiologic defense mechanism against various factors such as infection, burn, toxic chemicals, allergens, and other stimuli [2]. There are many components of an inflammatory response that participate in the associated symptoms and harmful effects to tissues. It involves a complex web of intracellular cytokine signals, which activate monocytes and/or macrophages releasing a variety of inflammatory mediators such as prostaglandins, platelet-activating factor (PAF), and arachidonic acid derivatives, which can originate locally or from cells that infiltrate in the site of inflammation [3]. Actually, nonsteroidal anti-inflammatory drugs (NSAIDs) are the most clinically important medicine used for the treatment of inflammation by inhibiting the cyclooxygenase (COX) pathway of arachidonic acid metabolism which produces prostaglandins [4]. Nevertheless, these drugs are limited in their effectiveness and cannot regulate the production of leukotrienes or PAF that continues to cause inflammation. Moreover, cyclooxygenase inhibitors could favor the appearance of thrombosis or renovascular hypertension in patients predisposed to these conditions [5]. The inhibition of phospholipase A_2_ (PLA_2_) may serve as a primary regulatory role in the development of inflammatory disorders and could deplete the sources of arachidonic acid and, therefore, its downstream metabolites and PAF, thus constituting an important strategy for the management of inflammatory disorders.

Chronic inflammation increases the risk of resistance and tumor recurrence, such as brain and breast cancer, indicating that eliminating inflammation may represent a valid strategy for cancer prevention and therapy [6]. Despite the advances in the field of anticancer drug discovery, the statistics are noteworthy; in 2012, 14.1 millions new cases of cancer were diagnosed worldwide, with 8.2 millions deaths [7]. Thus, there is still a necessity for the development of new therapies and the tumor microenvironment is an important source of multiple targets for cancer therapy, including oxidative stress and inflammation [7].

Nature has been a source of medicinal products for millennia, going along with the history of humanity. In chemotherapy field, around 75% of the anticancer agents used nowadays are derived from natural products of different origins, and plants are an important source of new promising therapies [7].* Phoenix dactylifera* L. (date palm) is an ancient plant used in folk medicine for the treatment of various diseases and disorders [2]. Dates and their constituents act as potent antioxidant, anti-inflammatory, and antitumoral and provide a suitable alternative therapy in various diseases cure [2]. According to the Inter Professional Fruits Group (GIFruits), dates production in Tunisia reached 246.000 tons in 2015, 40% of which is disposed or recycled as animal feed [8]. These are mainly dates attacked by pests, fermented and parthenocarpic dates (Sish). The previous study has shown that the aqueous ethanolic extract from* P. dactylifera* parthenocarpic dates contains several bioactive components such as p-coumaric acid hexose, rosmadial, quercetin, quercetin-3,7-di-O-glucoside, and ganodermic acid [8]. Therefore, the objective of this study was to evaluate the antioxidant, the anti-inflammatory, and the antiproliferative activities of the aqueous ethanolic extract from* P. dactylifera* parthenocarpic dates.

## 2. Materials and Methods

### 2.1. Extract Preparation

The aqueous ethanolic extract from* P. dactylifera* parthenocarpic date was prepared using previously described protocol [8]. Briefly, the dried powdered of healthy palm parthenocarpic dates (15 g) was macerated in 100 mL 71% ethanol solution (pH = 8.5) for 24 h at 59°C under stirring condition. The hydroalcoholic crude extract was filtered and concentrated under reduced pressure. The extract was dissolved in distilled water and then preserved at 4°C.

### 2.2. Storage Conditions

The dry extract was introduced into glass bottles protected (amber) from light and stored at - 20°C according to Del-Toro-Sánchez et al. [9]. Then, the extract stability was measured before the use of the aqueous ethanolic extract by determining total phenols, flavonoids, and tannins contents as described in the previously study [8].

### 2.3. Antioxidant Activity: DPPH Radical Scavenging Activity

DPPH radical scavenging activity of the aqueous ethanolic extract from* P. dactylifera* parthenocarpic dates was determined according to the method of Kim et al. [10]. The sample stock solution (100 mg of dry extract/mL) was diluted to final concentrations of 0.05, 0.1, 0.15, 0.2, 0.25, 0.3, 0.45, and 0.6 mg of dry extract/mL in ethanol. A total of 0.5 mL of 30 mM DPPH ethanol solution was added to 0.5 mL of sample solution at different concentrations and allowed to react at room temperature. After 30 min, the absorbance (A) was measured at 520 nm.

The ability to scavenge the DPPH radical was calculated using the following equation:

(1) Radical  Scavenging  capacity RSC, %=1−Asample−Asample  blankAcontrol x  100

where A_control_ is the absorbance of the control (DPPH solution without sample), A_sample_ is the absorbance of the test sample (DPPH solution plus test sample), and A_sample  blank_ is the absorbance of the sample only (sample without DPPH solution).

Results of DPPH radical scavenging were presented by IC_50_ value, defined as the concentration of extract which required reducing DPPH radicals by 50%.

### 2.4. Anti-Inflammatory Activity

#### 2.4.1. Animals and Experimental Design

Male albino mice of 20-25 g body masses, obtained from the Veterinary Research Institute (Sfax, Tunisia) and maintained under standard laboratory conditions (temperature 22 ± 2°C on 12 h light-dark cycle), were used in this study. Throughout the experimental period, the animals had* ad libitum* access to food and water. The experimental protocol was approved by the Medical Ethics Committee for the Care and Use of Laboratory Animals of the Pasteur Institute of Tunis (approval number: FST/LNFP/Pro 152012) and performed according to the European convention for the protection of living animals used in scientific investigations [11].

#### 2.4.2. In Vitro Study: Phospholipase A_*2*_ Inhibition Assay

Phospholipase inhibition assay was performed using PLA_2_/extract preincubation method during 1 h at room temperature in the absence of substrate [12]. Preincubation medium consisted of 10 units of PLA_2_ and the aqueous ethanolic extract from* P. dactylifera* parthenocarpic dates with varying concentrations from 0 to 200 *μ*g of dry extract/mL. A control sample was prepared accordingly without the aqueous ethanolic extract. The residual activity was measured titrimetrically at pH = 8 and 40°C with a pH-stat (Metrohm, Buchs, Switzerland), using 0.5% (m/v) egg yolk phosphatidylcholine as a substrate in 30 mL of 150 mM NaCl, 4 mM sodium taurodeoxycholate (NaTDC), and 8 mM CaCl_2_. The results were expressed as residual activity compared with the control. IC_50_ value, defined as the sample concentration (*μ*g/mL) at which 50% inhibition of the enzyme activity occurs, was calculated from the graph plotting enzyme residual activity against sample concentration. All tests were carried out for three sample replications and the results were averaged.

#### 2.4.3. In Vivo Study: Carrageenan-Induced Mice Paw Oedema

Mice were divided into three groups of six animals. Group 1 was served as a negative control and received saline water without the extract (solution of 0.90% (m/v) NaCl). Prior to the induction of the oedema, group 2 was injected intraperitoneally with the aqueous ethanolic extract from parthenocarpic dates (200 mg/kg), respectively. Group 3 was administered by an intraperitoneal injection of indomethacin (50 mg/kg) as a positive control. All drugs were administrated 30 min before the injection of carrageenan. Oedema was induced by injecting 0.3 mL of 2% carrageenan subcutaneously into the subplantar region of the left hind paw [13]. The paw oedema thickness was measured by using a digital micrometer (MT-045B; Shangai Metal Great Tools Co., Shangai, China) immediately before carrageenan injection and 1, 2, 3, and 4 h after carrageenan injection. Percentages of inhibition of inflammation were obtained for each group using the following ratio:

(2) Inhibition %=Vt−V0control−Vt−V0treated×100Vt−V0control

where V_t_ is the average volume for each group at different hours after treatment and V_0_ are the average volume obtained for each group before any treatment.

### 2.5. Antitumoral Activity

#### 2.5.1. Cell Line and Culture Conditions

The human tumor cell lines MDA-MB-231 (breast carcinoma) and MCF-7 (breast adenocarcinoma) were a kind gift of Dr. Khadija Essafi-Benkhadir, Institute Pasteur Tunis, Tunisia. Tumor cells were routinely maintained in Dulbecco's modified Eagle's minimum essential medium (DMEM) supplemented with 10% heat inactivated fetal bovine serum, 2 mM glutamine, 1% penicillin, and streptomycin at 37°C in a humidified atmosphere containing 5% CO_2_.

#### 2.5.2. Cell Viability Assay

The effect of the aqueous ethanolic extract from* P. dactylifera* parthenocarpic dates on the viability of MDA-MB-231 and MCF-7 cells was assessed using the MTT (3-(4,5-dimethylthiazol-2-yl)-2,5-diphenyltetrazolium bromide) method according to Boulaaba et al. [14]. Tumor cells at optimal density were seeded in 96-well microplates (Nunc™ 96-Well Microplates-Thermo Scientific) and incubated overnight at 37°C under 5% CO_2_ to allow them to attach. Aqueous ethanolic extract from* P. dactylifera* parthenocarpic dates at different concentrations (1 to 100 mg of dry extract/mL) was added to adhered cells and incubated for 24 h and 72 h. After the indicated time, MTT solution was added and cells were incubated for a further 4 h. The solutions were aspirated out, and DMSO was added to solubilize the formazan crystals within metabolically viable cells. Absorbance was determined by a microplate reader at 560 nm on a multidetection microplate reader (Thermo Labsystems, Franklin, MA, USA). Results were evaluated by comparing the absorbance of the treated cells with the absorbance of wells containing cell treated by the solvent control. Conventionally, cells incubated only with the medium and the solvent were considered the control with 100% viability.

All experiments were performed at least twice in triplicate. The concentration of the substance required for 50% growth inhibition (IC_50_ value) was estimated as that resulting in 50% decrease in absorbance as compared to control incubated simultaneously without test substances.

### 2.6. Statistical Analysis

Values were expressed as the mean of triplicate analysis of the samples (n = 3) standard deviation (± SD). Statistical analysis was performed by one-way analyses of variance (ANOVA) and unpaired Student's t-test was used to determine significant differences in hyperglycemia and where appropriate. Differences were considered statistically significant if p < 0.05.

## 3. Results and Discussion

### 3.1. DPPH Radical Scavenging Activity

**Figure 1:**
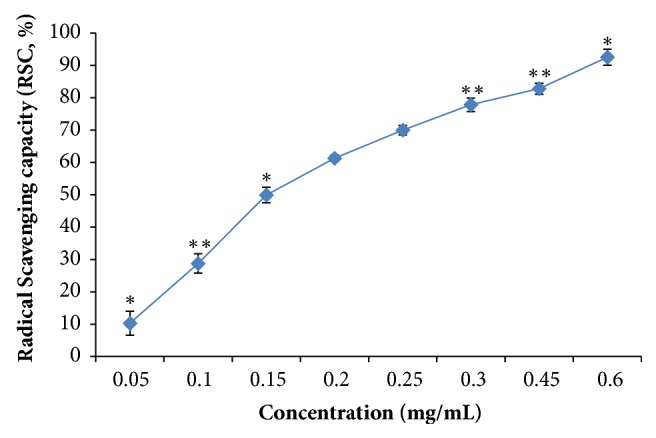
Antioxidant activities of the aqueous ethanolic extract from* Phoenix dactylifera *parthenocarpic dates. Differences in antioxidant activity were estimated by one-way analyses of variance (ANOVA) and unpaired Student's t-test compared to the control (*∗*p <0.05 and *∗∗*p <0.01).

The antioxidant activity was defined as the mean of free radical scavenging capacity. This activity was measured using the 1,1-diphenyl-2-picrylhydrazyl free radical (DPPH), which is a stable free radical and in the presence of the total extract; it was scavenged. In this study, the antioxidant effect of the aqueous ethanolic extract from* P. dactylifera* parthenocarpic dates was examined by DPPH radical scavenging capacity. The aqueous ethanolic extract showed a potential antioxidant activity in DPPH radical scavenging with an IC_50_ value of 0.15 ± 0.011 mg of dry extract/mL (p < 0.05) (Figure 1). The aqueous ethanolic extract was shown to scavenge 94% of superoxide radicals at 0.6 ± 0.03 mg/mL (p < 0.05). This extract had a strong free radical scavenging ability compared to the aqueous date extract (0.8 mg/mL) [15] based on the same method. Moreover, the aqueous ethanolic extract from parthenocarpic dates exhibited high DPPH scavenging activity compared with* Diplotaxis simplex* extract (0.4 mg/mL) [16] and* Aloe vera *leaf (0.635 mg/mL) [12]. These results were in line with those of Mansouri et al. [17] and Hasan et al. [18] who reported that date fruits exhibited potent DPPH scavenging capacities.

Fruits contain different antioxidant compounds. Therefore, measuring the antioxidant capacity of each compound individually becomes very difficult. Several methods have been developed to estimate the antioxidant potential of different plant materials. Usually, these methods measure the ability of antioxidants to scavenge specific radicals. The present study showed that the aqueous ethanolic extract had significant antioxidant activity toward the DPPH free radical assay. The antioxidant activity could be due to the high content of the phytochemical compounds of the aqueous ethanolic extract from parthenocarpic dates. In fact, the content of total phenols was estimated at 513 ± 2 mg of GAE/g of the dry extract [8] and the extract maintains approximately up to 95% stability in phenolic content when stored at −20°C in the dark. Moreover, the physicochemical results by liquid chromatography-tandem mass spectrometry (LC–MS/MS) analysis demonstrated that the aqueous ethanolic extract from* P. dactylifera* parthenocarpic dates contain several bioactive components acting as antioxidants, such as p-coumaric acid hexose, rosmadial, quercetin, and ganoderenic acid [8]. The antioxidant activity of phenolic compounds is a result of their redox properties, which can play an important role in absorbing and neutralizing free radicals [19].

Reactive free radicals, such as superoxide anion radical, hydroxyl radical, and hydrogen peroxide, have been implicated in the development of many diseases such as cancer, coronary heart disease, autoimmune disease, diabetes, sclerosis, cataracts, and chronic inflammation. According to the important activity on free radical scavenging, the anti-inflammatory effect of the aqueous ethanolic extract from* P. dactylifera* parthenocarpic dates was investigated* in vitro* and* in vivo*.

### 3.2. Evaluation of the Anti-Inflammatory Activity

#### 3.2.1. In Vitro Study: Inhibition of Phospholipase A_2_ Activity

Phospholipase A_2_ is a class of enzyme that catalyzes the hydrolysis of membrane phospholipids releasing arachidonic acid, which serves as a substrate for proinflammatory mediators, such as prostaglandins and leukotrienes. The design of specific inhibitors for PLA_2_ might help in the development of new anti-inflammatory drugs.

**Figure 2:**
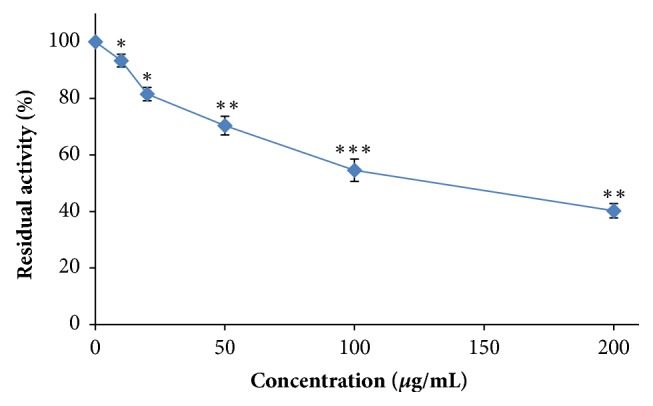
Effect of the aqueous ethanolic extract from* Phoenix dactylifera *parthenocarpic dates on phospholipase A_2_ activity (*∗*p <0.05, *∗∗*p <0.01, and *∗∗∗* p < 0.001).

In this study, the aqueous ethanolic extract was evaluated for their inhibitory effect on the proinflammatory PLA_2_ activity. The* in vitro* assay results revealed that the aqueous ethanolic extract showed a significant inhibitory activity against PLA_2_ activity depending on the doses of the extract (Figure 2). At a concentration of 130 *μ*g of dry extract/mL, the* P. dactylifera* extract reduced by 50% the activity of the enzyme in a highly significant manner (p < 0.001). This extract exhibited a potent inhibition of PLA_2_ than the* Aloe vera* L. extract (IC_50_ = 0.22 mg/mL) [12],* Diplotaxis simplex* extract (IC50 = 2.97 mg/mL) [16], and* Polygonum multiflorum* extract (IC_50_ = 680 *μ*g/mL) [20]. The PLA_2_ inhibition could be explained by the richness of the aqueous ethanolic extract in phenolic compounds, such as flavonoids, which are able to inhibit key enzymes related to inflammation process [12]. Besides, molecular modeling studies suggested that phenolic hydroxyls are linked to the amino acid Asp 49 of PLA_2_ and influence the capacity of this residue to participate in the coordination of the calcium atom, that is, essential to the catalytic activity [21].

#### 3.2.2. In Vivo Study: Carrageenan-Induced Mice Paw Oedema

The carrageenan-induced paw oedema is a well-known acute model of inflammation that is widely used for screening novel anti-inflammatory compounds. Immediately after carrageenan injection, there is a cascade of mediators' release, as histamine, serotonin, bradykinin, and PLA_2_. These mediators promote an increase in vascular permeability and signal for arachidonate metabolites and nitric oxide release, until the 6th hour.

Before induction of the inflammatory response, mice paw thickness was found to be 0.20 ± 0.01 cm (p < 0.05). The phlogistic agent when injected locally into the rat hind paw of the control group induced a severe inflammatory reaction, characterized by an increase in paw thickness up to 0.27 ± 0.01 cm (p < 0.05) and 0.32 ± 0.01 cm (p < 0.01) after 1 and 4 h, respectively (Table 1). The maximum peak was observed 3 h after injection when the mice paw thickness was found to be 0.36 ± 0.01 cm (p < 0.05) (Table 1). The treatment with the standard indomethacin significantly inhibited the paw thickness of carrageenan-induced mice, which reached 0.26 ± 0.01 cm and 0.22 ± 0.02 cm after 3 h and 4 h, respectively (Table 1). As shown in Table 1, the aqueous ethanolic extract from parthenocarpic dates showed significant anti-inflammatory activity when administered intraperitoneally, in the carrageenan-induced rat paw oedema test. The paw thickness of carrageenan-induced mice showed a decrease after 1 h (0.22 ± 0.02 cm (p < 0.05)) stronger than the indomethacin effect (0.24 ± 0.01 cm (p < 0.05)). The percentage of inhibition of oedema by the aqueous ethanolic extract, 3 h after carrageenan injection, ranged from 93% to 100%, whereas the reference drug produced 75% of inhibition after 3 h (Table 1). These results confirmed the anti-inflammatory activity of the aqueous ethanolic extract from* P. dactylifera* parthenocarpic dates, which could lend support for its pharmaceutical use. Similar results were observed with genus* Cystoseira* extract [1] and* Diplotaxis simplex* extract [16] which reduced the paw oedema in mice, after carrageenan injection. Moreover, Mohamed and Al-Okbi demonstrated that oral administration of methanolic or aqueous extracts of edible portion of* P. dactylifera* dates suppressed the inflammation in the foot of adjuvant arthritis rats [22].

Carrageenan rat paw oedema test produced an acute inflammation that results from the sequential action of several mediators. Histamine and serotonin were mainly released during first 1.5 h after carrageenan injection, kinin was released until 2.5 h, and at the last step, inflammation was continued until 5 h by prostaglandins. The aqueous ethanolic extract inhibited the PLA_2_ enzymatic activity in the first phase of inflammation (arachidonate metabolites generation). The bioactive compounds, especially quercetin, present in the aqueous ethanolic extract can be the responsible for the anti-inflammatory activity [23]. In addition, the antioxidant potential observed in* P. dactylifera* extract can be also contributed for reducing inflammation. Thus, the potent anti-inflammatory activity of* P. dactylifera* parthenocarpic dates extract may be related to cumulative effects of different active compounds to reduce the synthesis, release, and action of prostaglandins or free radicals.

Increasing scientific evidence shows that polyphenols are good antioxidants and are effective in preventing inflammatory diseases and can also be used as chemopreventive agents for cancer. These molecules might act as cancer blocking agents, preventing initiation of the carcinogenic process and as cancer suppressing agents, inhibiting cancer promotion and progression [1]. Herein, we evaluated the anticancer effects of the aqueous extract on human breast cancer (MDA-MB-231 and MCF-7) cells* in vitro*.

### 3.3. Evaluation of the Antitumoral Activity

Breast cancer is the most common and leading cause of cancer-related mortality among women globally [24]. In 2012, more than 464,000 new cases were diagnosed with breast cancer in the European Union (EU) and in the United States of America (USA) [24]. It was estimated that there will be 249.260 new cases of female breast carcinoma in the year 2016 [24]. This has led to an increased interest and active search for novel anticancer agents from natural products. In the present study, we attempt to evaluate the anticancer effects of the aqueous ethanolic extract from* P. dactylifera* parthenocarpic dates on human breast carcinoma (MDA-MB-231) and adenocarcinoma (MCF-7) cells.

**Figure 3:**
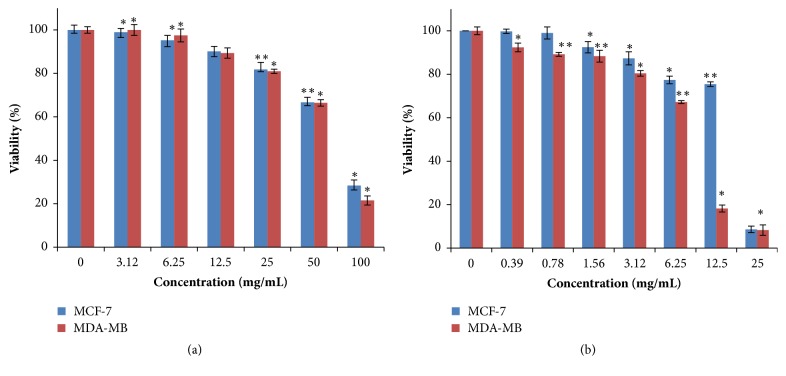
Effect of the aqueous ethanolic extract from* Phoenix dactylifera *parthenocarpic dates on MDA-MB-231 and MCF-7 cancer cell lines viability at 24 h (a) and 72 h (b). Differences between selected groups were compared to nonparametric analysis of variance (ANOVA) with Bonferroni post hoc multiple comparison test compared to the control (*∗*p <0.05 and *∗∗*p <0.01).

The effect of different concentrations (1 to 100 mg of dry extract/mL) of the aqueous ethanolic extract on tumor cell viability using the MTT method was assessed (Figure 3(a)). After 24 h incubation of MDA-MB-231 cells with the aqueous ethanolic extract, no significant inhibition of cell growth was observed at (1-25 mg/mL). However, results showed that the cytotoxic effect appeared at high concentrations (50 and 100 mg/mL), when cell viability decreased by 43.66% and 78.48%, respectively (Figure 3(a)). Similarly, results have been observed after treatment of MCF-7 with the aqueous ethanolic extract for 24 h. MCF-7 cells growth inhibition and signs of cytotoxicity were not significantly apparent until a concentration of 25 mg/mL (80% of cell viability). Then cell viability diminished with the dose of 50 and 100 mg/mL to 43.34% and 71.67%, respectively (Figure 3(a)). Interestingly, the viability of both MDA-MB-231 and MCF-7 tumor cells decreased drastically in a dose-dependent manner after 72 h of treatment with aqueous ethanolic extract with respective IC_50_ values of 8 ± 0.02 mg/mL (p < 0.01) and 18 ± 0.02 mg/mL (p < 0.01) (Figure 3(b)).

MDA-MB-231 and MCF-7 cells viability inhibition was more pronounced after 72 h of incubation as compared to 24 h of aqueous ethanolic extract treatment. This result suggests that the inhibition of cell viability may be related to the suppression of proliferation rather than to any cytotoxic or cytolytic effects. This activity might be due to the presence of specific compounds in parthenocarpic dates, like flavonoids glucosides (quercetin), which are known by their cancer chemoprotective attributes [25]. In this context, Rahmani et al. [26] confirmed the therapeutic effects of* P. dactylifera* dates in preventing against cancer are due to its richness in polyphenolic compounds. In addition, previous studies showed that polyphenolic compounds affect cancer cell growth by inducing apoptosis in many cell lines related to breast cancer [24]. In addition, the anticancer effect of the methanolic extract of Ajwa date on human breast adenocarcinoma (MCF-7) cells was evaluated* in vitro*. MCF7 cells treated with concentrations (5, 10, 15, 20, and 25 mg/mL) of methanolic Ajwa date extract inhibited the growth and the proliferation of its cells by inducing cell cycle arrest. It also induced MCF7 cell death via apoptosis in a dose and time-dependent manner by the activation and changes in genetic expression associated with apoptosis [24].

One important aspect of carcinogenesis is recognized to be the involvement of inflammation. Many studies have been conducted to treat cancers by using various anti-inflammatory agents. In fact, the use of anti-inflammatory agents can alter cancer cell and its microenvironment, potentially increasing apoptosis, and decreasing migration. Interestingly, natural phenolics could exhibit anti-inflammatory properties and also a possible role in the inhibition of cancer development through a number of basic cellular mechanisms [27].

## 4. Conclusion

*P. dactylifera* plays an important role in social, economic, and ecological Tunisian sectors. Some date palms produce parthenocarpic fruit named Sish. This study revealed that the aqueous ethanolic extract from* P. dactylifera* parthenocarpic dates provides a strong antioxidant activity associated with an interesting anti-inflammatory activity and significant antiproliferative activity against MDA-MB-231 and MCF-7 cancer cell lines. The antitumoral effect of aqueous ethanolic extract could be attributed to a single molecule or to the synergic activity of several components of the total extract.

## Figures and Tables

**Table 1:** Anti-inflammatory effect of the intraperitoneal administration of the aqueous ethanolic extract from *Phoenixdactylifera* parthenocarpic dates in Carrageenan-induced mice paw oedema test.

**Treatment**	**Paw thickness (cm)**				**Inhibition (**%**)**			
	**1 h**	**2 h**	**3 h**	**4 h**	**1 h**	**2 h**	**3 h**	**4 h**
**Control**	0.27 ± 0.01*∗*	0.32 ± 0.02*∗∗*	0.36 ± 0.01*∗*	0.32 ± 0.01*∗∗*	-	-	-	-
**Indomethacin**	0.26 ± 0.01*∗*	0.26 ± 0.01^ns^	0.24 ± 0.01*∗∗*	0.22 ± 0.02*∗*	14.28	50	75	83.33
**Extract**	0.22 ± 0.02*∗*	0.24 ± 0.02*∗*	0.21 ± 0.01*∗*	0.2 ± 0.02*∗*	71.2	66.66	93.75	100

Values are expressed as mean ± SD (n=6 animals).

*∗*: differences were considered statistically significant if p <0.05.

*∗∗*: differences were considered statistically significant if p <0.01.

ns: not significant.

## Data Availability

The data used to support the findings of this study are available from the corresponding author upon request.

## Additional Points

(i) The aqueous ethanolic extract showed a potential antioxidant activity. (ii) The* in vitro* study showed that the extract inhibited the phospholipase A_2_. (iii) The* in vivo* study showed a significantly decrease in the paw oedema in mice. (iv) The extract was effective in inhibiting MDA-MB-231 and MCF-7 cancer cell growth.
